# Feasibility Studies of Charge Exchange Measurements in pp Collisions at the LHC

**DOI:** 10.3390/e24091188

**Published:** 2022-08-25

**Authors:** Anna Fehérkuti, Gábor I. Veres, Ralf Ulrich, Tanguy Pierog

**Affiliations:** 1 Institute of Physics, Eötvös Loránd University (ELTE), 1053 Budapest, Hungary; 2 Institute for Astroparticle Physics, Karlsruhe Institute of Technology (KIT), 76049 Karlsruhe, Germany; 3 Modular Mind Labs GmbH, 76137 Karlsruhe, Germany

**Keywords:** charge exchange reaction, forward neutron, energy spectrum, ROC curve, Compact Muon Solenoid (CMS), zero degree calorimeter (ZDC), hadron forward calorimeter (HF), generator-level simulation, Cosmic Ray Monte Carlo (CRMC), muon puzzle

## Abstract

(1) Pions produced in the development of extended atmospheric cosmic ray air showers subsequently decay to muons. The measured yield of those muons is generally underestimated by current phenomenological models and event generators optimized for cosmic ray physics. The importance of those disagreements motivates the feasibility studies for testing these models at the Large Hadron Collider (LHC) energies, at the highest center-of-mass energies achievable in a laboratory. The interaction of a nucleus and a virtual pion created in a charge exchange reaction at the LHC is a similar process to those contributing to the development of air showers in case of cosmic rays. The crucial problem of such an analysis is the selection of charge exchange events with the highest possible efficiency and high purity from proton–proton collisions at the LHC. (2) For this we consider distributions of various measurable quantities given by event generators commonly used in cosmic ray physics. (3) We examine the expected distributions of energy deposited in different calorimeters of an LHC experiment. We consider the geometrical acceptance and energy resolution of the detectors at the Compact Muon Solenoid (CMS) experiment, as an example. We determine a working point cut from the various options for event selection, and compare signal and background predictions using different models for a representative simple observable, such as average transverse momentum or charge particle yield. (4) A set of event selection cuts along these considerations is proposed, with the aim of achieving optimal efficiency and purity.

## 1. Introduction

Cosmic rays consist of high energy particles (90% protons, 9% helium nuclei, 1% other nuclei, even uranium [1]) arriving from the Universe, reaching the Earth. In a broader concept, we can call the constituents of cosmic rays (stellar and solar) particles too, such as electrons, positrons, neutrinos and photons, along with secondary particles which are created when the incident particles collide with the atmosphere, and their decay daughters.

The Pierre Auger Observatory (PAO) [2] was built for the examination of cosmic rays. Here, the most recent and frequently used models simulating particle collisions do not reproduce the measured data for the number of cosmic muons [3,4,5,6], which is measured with their surface detectors. This is called the *muon puzzle*, which also motivated the work presented here: it is necessary to understand muon (or charged pion) production more qualitatively in the laboratory, even if the center-of-mass energy at the LHC is lower than that of the highest energy cosmic rays to test these phenomenological predictions.

In high energy hadron collisions, the so-called *charge exchange reaction* may appear, in which a proton turns into a neutron emitting a virtual reggeon [7,8], likely a pion. That pion can interact with another proton, creating a new particle. In cosmic rays, the collision of a pion with a nucleon (in the nuclei of the molecules in the air) is very similar to a collision of a virtual pion with a nucleon of a nucleus in a charge exchange reaction at the Large Hadron Collider (LHC). That is why the charge exchange reactions are studied with the frequently used Monte Carlo (MC) simulations of collision events in the following chapters.

## 2. Materials and Methods

The authors of some of the event generators considered here indicated the possibility to include charge exchange reactions in the Cosmic Ray Monte Carlo (CRMC v1.7) [9], which is a widely used event generator in cosmic ray physics. The present article contains generator-level information provided by this version of CRMC. The charge exchange reaction has a cross-section at the level of millibarns (1 b (barn) = 10^−28^ m^2^), whereas that of inelastic scattering is around 70 mb at LHC energies [10].

The fraction of inelastic events that involve a charge exchange is predicted to be about 1.5% [7,8]. Actually, there are more types of charge exchange: instead of a pion, a ϱ or $a_{2}^{+}$ can be exchanged with a one-hundred-times smaller cross-section ($\sigma_{\varrho^{+}}<\sigma_{a_{2}^{+}}<<\sigma_{\pi^{+}}$), and one can also consider multiple exchanges. Different Monte Carlo models (EPOS 1.99, EPOS-LHC, PYTHIA 6.4.28 and SIBYLL 2.3c [11]) use different sets of virtual particles, and produce charge exchange technically only in one beam direction (the neutron always propagates in the positive direction), which arrives at the zero degree calorimeter on that side, and is therefore denoted by “ZDC+”.

During the analysis, generator-level inclusive proton–proton collisions were examined at a center-of-mass energy of 7 TeV. The number of generated events was 4 million in the case of the EPOS 1.99 model, and for the comparison to other models, 100 thousand events in each case. Although LHC can provide both pp and pPb collisions to the experiments, proton–proton collisions are foreseen for the beginning of the LHC Run 3; therefore, this paper discusses the proton–proton collision system.

The Compact Muon Solenoid (CMS) [12] is one of the four large experiments at the LHC, featuring zero degree calorimeters that are able to detect neutrons, and we have chosen to illustrate the feasibility of measuring charge exchange reactions by considering crudely the acceptance, resolution and geometry of CMS detectors. Three of the subdetectors of CMS would be useful for the event selection optimized for charge exchange, as described below.

**ZDC**       The most important detector from the point of view of a charge exchange event is the *zero degree calorimeter* (ZDC), because the charge exchange neutron (the neutron that was created from the initial proton via charge exchange) is absorbed in the ZDC; in the |η|>8.4 angular region (pseudorapidity is $\eta =-\log\left(\tan(\vartheta /2)\right)$, where ϑ is the polar angle, measured from the beam direction denoted by the *z* axis). It is a relatively small (92×(711+116)×705 mm [13]) sampling Cherenkov calorimeter located 140 m (in the beam direction) from the interaction point on both sides, so it can measure only electrically neutral particles (all charged ones are already deflected due to the large magnetic field in the LHC dipole magnets). In case of CMS, the active detector mass of the ZDC is quartz, whereas the absorber is made of tungsten. Those ZDCs have an electromagnetic (EM) section closer to the interaction point, followed by a hadronic (HAD) one.
**HF**          Another important sampling calorimeter in the CMS experiment in the forward region is the *hadron forward calorimeter* (HF). Its acceptance is the 3<|η|<5.2 interval (HFs take place on both sides of the interaction point, too), comprising 18 segments made of iron and quartz.
**Tracker**  The tracker system of the CMS experiment consists of the silicon pixel and silicon strip detectors, and the total angular coverage of the *tracker* is |η|<2.5.

## 3. Results

### 3.1. Selection on Energy

#### 3.1.1. Energy in the Zero Degree Calorimeter

**Figure 1:**
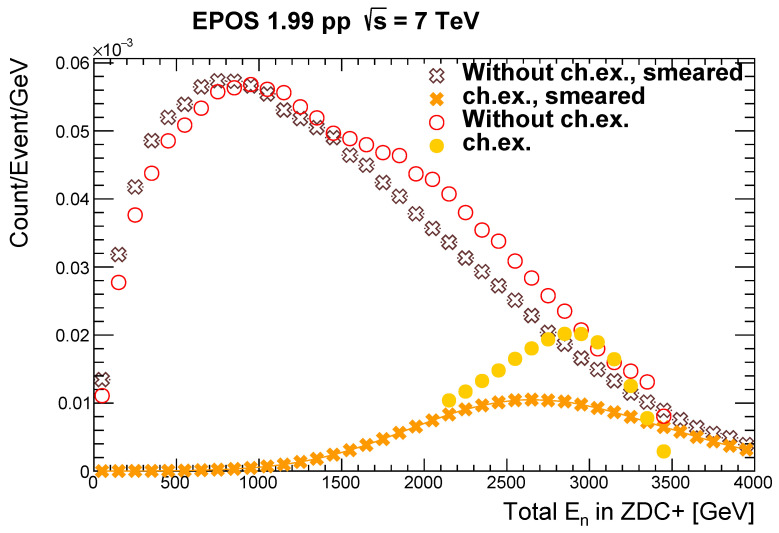
Energy distributions with generator-level values (circles) and smeared energy values (crosses) for neutrons arriving at the ZDC+.

When examining the distributions of energy deposited by neutrons in the ZDC+ in charge exchange events and inclusive ones excluding charge exchange, one can see that they follow characteristically different shapes (Figure 1). Note here that the discontinuity at 2100 GeV does not reflect a physical bound; it is only due to the features of the model employed here that limit the momentum transfer, and it practically disappears when the calorimeter energy resolution smears the generator-level energy values. According to an analysis on the ZDC energy resolution [14], the resolution is at most 25%. Using this value, one can apply Gaussian smearing on the energy distribution. In Figure 1, one can see the smeared distribution in comparison to the original one (using the generator level values). Even though the relative resolution was kept constant at 25%, the energy dependence of the absolute resolution led to the inflexion in the smeared distribution.

**Figure 2:**
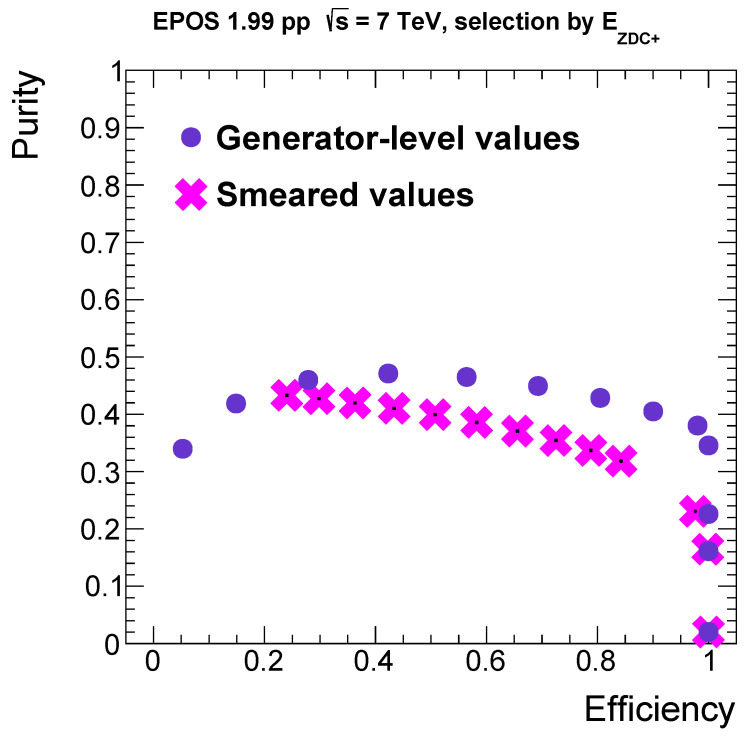
ROC curve for the energy selection on the smeared energy distributions, compared to the original, generator-level one.

The energy of the charge exchange neutrons is similar to the energy of the initial proton, 3500 GeV; thus, for energies closer to the beam energy, charge exchange events have larger contributions. By making use of this property, one can determine a requirement for the minimal energy deposition in the detector that can be used as a selection cut. On the basis of the distributions in Figure 1, we selected ten different minimal energy cuts between 0 and 3300 GeV and considered two quantities for the characterization of these selections (lower bounds), as follows. *Purity* expresses the fraction of the number of charge exchange events with respect to all events above the energy cut, and *efficiency* denotes the fraction of the number of selected charge exchange events with respect to all the charge exchange events. By plotting these quantities for each region, one obtains the so-called receiver operating characteristic (ROC) *curve*. The ROC curve for the smeared distributions, taking into account the energy resolution, can be seen on Figure 2. When comparing this to the generator-level one, a suppression in purity is visible, as expected.

Note here that multiple points are placed at efficiency = 1; those are a result of the aforementioned discontinuity feature in the generated neutron energy distribution. Furthermore, this ROC curve is rather flat and has no points near the corner (1, 1), which would be the ideal case. Nonetheless, one can choose a working point optimally at high efficiencies. A clear best choice for the working point is at (0.98, 0.38) in the non-smeared case, which means that one has to require a minimum of 2140 GeV total energy deposited by neutrons in ZDC+ as the first step of the event selection.

#### 3.1.2. Energy in the Hadron forward Calorimeter

**Figure 3:**
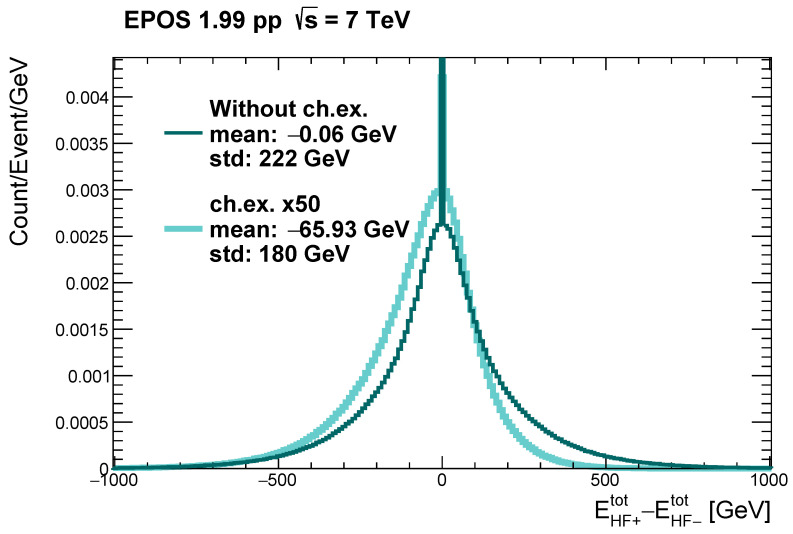
Distribution of the difference in the total energy entering the geometrical acceptance of the HF calorimeters on the positive and negative sides of the CMS experiment. The distribution for the charge exchange events was multiplied by a factor of 50 for better visibility.

In order to further improve the selection, one can make use of the asymmetry of the total energy deposited in the hadron forward calorimeters on the two sides of the interaction point. Some asymmetry should be intuitively expected from momentum conservation, since the charge exchange neutron carries a large momentum in the beam direction. The difference between the total energy deposited (by all hadrons in a given event) in the HFs on the positive and on the negative side is calculated, and the distribution of the difference is plotted in Figure 3.

One can see that this distribution is indeed asymmetric for charge exchange events, but on average symmetric for the others. This can serve as another distinction between charge exchange and inclusive events. Since the cross-sections of charge exchange events are small with respect to the inelastic ones, it is necessary to make a cut first on the ZDC energy (requiring a minimum of 2140 GeV in the ZDC+) and only then consider the HF energy asymmetry.

The apparent asymmetry in the charge exchange case is a consequence of the fact that the simulation produces charge exchange neutrons only in one direction; thus, the average over the event ensemble does not symmetrize it. One has to keep in mind that in experimental circumstances the neutron can propagate in both directions, so one has to consider ∣Etot_HF+_ − Etot_HF−_∣ instead of simply “Etot_HF+_ − Etot_HF−_” at the selection step.

#### 3.1.3. Minimal Energy in the Hadron Forward Calorimeter

One can make a further observation when studying Figure 3, namely, that at zero energy difference there is a sharp peak in each of the otherwise smooth distributions. This peak originates from events with no particles propagating in the direction of any of the HFs. Thus, by requiring some minimal energy (10 GeV in this case) in at least one of the geometrical acceptance regions of the HFs, it is possible to remove many background (i.e., non-charge-exchange) events. Quantitatively, 1.2% of the charge exchange events and 4.1% of the inclusive (without charge exchange) events are discarded with this cut—a nearly four times larger fraction of background events.

**Figure 4:**
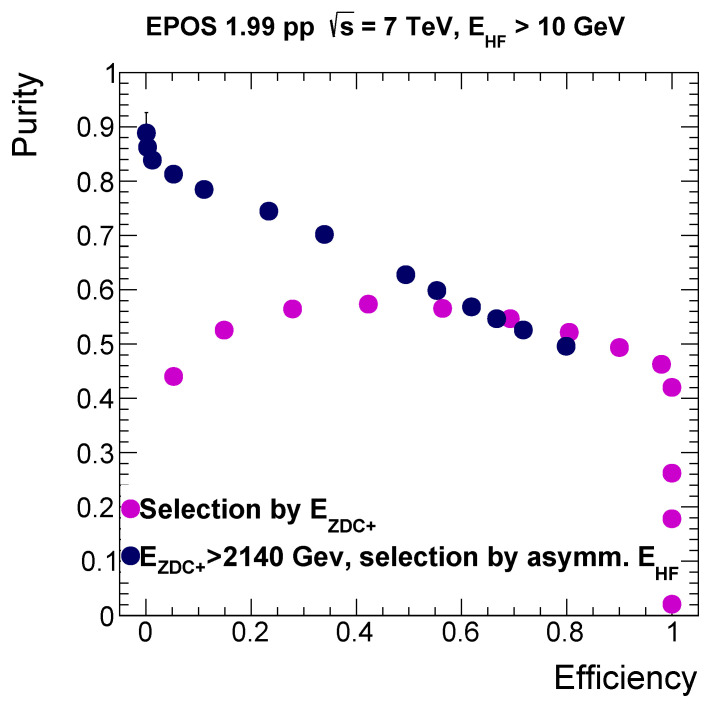
ROC curve corresponding to the selection on the HF energy difference (dark blue dots), where a minimal energy of 2140 GeV propagating to the ZDC+ acceptance is required, together with a minimal energy in any of the HF acceptances; and the ROC curve requiring only a minimal energy in any of the HFs and using the selection on the ZDC energy (purple dots).

A new ROC curve could be produced for this more complex selection method, (dark blue dots on Figure 4), where at first events in which less than 2140 GeV energy was deposited in the ZDC+ and in which there was no energy deposit in any of the HFs were removed. Technically, an upper threshold of 10 GeV was required to declare that there was no energy propagating to the HFs. After that, various lower cuts on the absolute value of the positive minus the negative side total energies in the HFs were chosen, and the ROC curve was plotted using the purity and efficiency of those selections.

We can compare the ROC curve obtained this way to the previous one, where only minimal energy in any of the HFs was required and the selection on the ZDC energy was used. Note that the efficiency was redefined; we do not have any higher efficiency values than ≈ 0.8, due to the difference in the basis of the comparison, since we already applied selections before studying the effect of the HF energy asymmetry cut. We were able to reach significantly higher purity values with this new selection method.

### 3.2. Bias Tests

**Figure 5:**
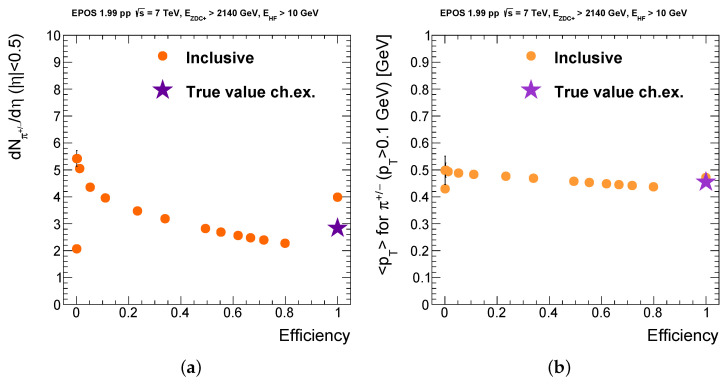
(**a**) Pseudorapidity density of charged pions in the tracker as a function of event selection efficiency for different working points. (**b**) Average transverse momentum of charged pions (above 0.1 GeV) as a function of efficiency.

It is important to see how the selection method biases observable quantities, and minimizes the selection bias. Ideally, the observables to be measured in a real experiment are not, or only minimally biased by the event selection and the chosen working point. The event selection cuts employ the information from the ZDC and from the HF; therefore, we consider observables in a different pseudorapidity range, for example, in the geometrical acceptance of the CMS tracker system. We can consider, for an example quantity, the distributions of charged particles or charged pions, namely, their pseudorapidity density (yield) in the |η|<0.5 region and their average transverse momentum in full tracker acceptance |η|<2.5, where the average is applied to particles with *p_T_* > 0.1 GeV, taking into account the approximate acceptance of the tracker in *p_T_*. Our aim is to have a set of events that resembles the charge exchange events as closely as possible after the event selection, or at least, to minimize the apparent selection bias. Figure 5 shows the average dN/dη and *p_T_* quantities as a function of efficiency, for various working points, and we also plot the true values of these quantities for the charge exchange events taken from the event generator with no further selection (depicted by star markers). This way, one can choose the final working point such that the selected dataset approximates the true generator-level values.

The point with the highest efficiency corresponds to the value before any cuts; the others in decreasing order of efficiency: the first one is obtained with the ZDC energy cut and minimal HF energy cut; after that, the one with the loosest cut on the HF-asymmetry (in which case, actually no events are discarded yet); and moving to the left, values corresponding to ever stricter cuts.

Generally speaking, while the average transverse momentum of charged pions in the tracker only slightly varies when taking different working points, the pseudorapidity density shows stronger dependence. That is expected because requiring a larger energy difference in the HFs correlates with the amount of total energy in the HFs, and in turn, with a larger charged particle multiplicity.

According to Figure 5, choosing a new working point at efficiency = 0.49, with purity = 0.63 (with respect to Figure 4), the selected dataset reproduces the true value of the pseudorapidity density for charge exchange events, and at the same time, it gives only 1% less than the true value of the average transverse momentum. This way, we obtained an event selection requiring a minimum of 2140 GeV energy propagating into the ZDC+ acceptance, a minimal energy of 10 GeV for the acceptance of any of the HFs and also a larger than 102 GeV energy difference between the energy of particles falling into the acceptance of the HFs on the two sides of the CMS. This selection is model dependent, but for the EPOS 1.99 generator, it resulted in the closest values of the examined quantities compared to those of the true charge exchange events. Furthermore, it is noticeable that the purity was almost doubled with respect to the selection only on the ZDC energies (see Figure 2, where it was around 0.38).

### 3.3. Model Dependence

It is important to repeat these studies for other models, since our selection cuts that resulted in an efficiency of 49% and 63% purity, are based so far only on the EPOS 1.99 event generator. Other models take into account different processes; thus, their parameters and their physics content may vary. Parameters are fixed generally by a many-parameter fit to various datasets, so one commonly uses the default settings. Our investigations also revealed the features of the actual models studied. We generally assume that true (to be measured) values of the considered quantities lie in the range spanned by the predictions of various models.

**Figure 6:**
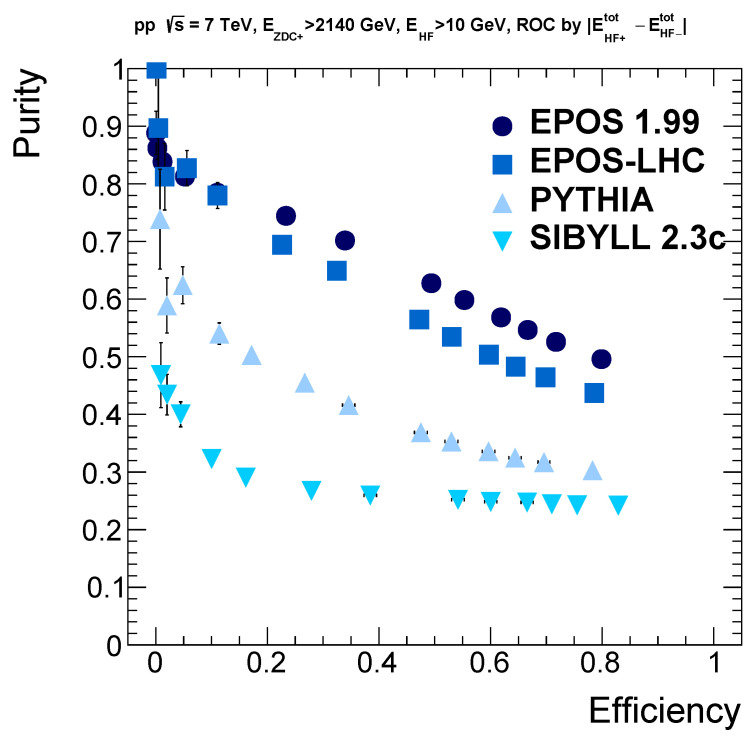
ROC curves for the cuts in HF energy difference for the four models that can handle the charge exchange processes. A minimal energy deposit from neutrons in the ZDC+ is required in addition, together with a minimal energy in any of the HF calorimeters.

Nevertheless, these predictions provide an estimate of reality, and we concentrate on the most important quantities from the above analyses. We are mostly interested in the final ROC curves (when one requires a minimum of 2140 GeV hadronic energy in the ZDC acceptance and at least 10 GeV energy in the HF acceptances together). We examined the purity and efficiency of event sets after certain cut values for the energy difference between the HFs. One can see the obtained ROC curves for all the four models that can handle the charge exchange processes in Figure 6. The two different tunes of EPOS give similar shapes for the distributions. PYTHIA and SIBYLL 2.3c produced a different shape for the curve and much less promising predictions too; according to them, the purity values are quite low. The variability of these model predictions already highlights the value of any possible experimental measurements that could be carried out in this corner of high-energy physics.

**Figure 7:**
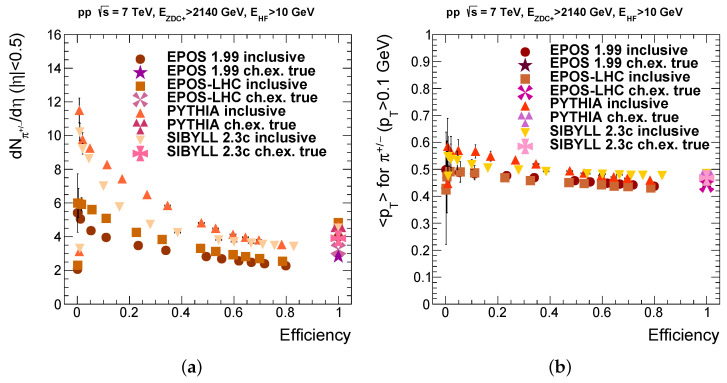
(**a**) Pseudorapidity density of charged pions in the tracker for different possible working points. (**b**) Same for the average transverse momentum, taking into account that tracker acceptance in transverse momentum.

Furthermore, it is also necessary to repeat the analysis of the bias tests for the other models that can handle charge exchange, as presented on Figure 7. A similar pattern can be seen in these figures; the EPOS tunes behave similarly, whereas the other two models give more widely varying predictions (see in Table 1). However, concerning the aspect of the working point (in)dependence they agree better. While the pseudorapidity density strongly depends on the actual cut, the average transverse momentum can be regarded as (roughly) independent of it.

As one can see in the last column of Table 1, concerning purity there was a wide range of values suggested by the models for the working point. We chose to define these final working points by matching the average dN/dη value for the selected set of events to the true value of the dN/dη in charge exchange events given by the interaction model (event generator) without any selection (bias). The reason for this choice was that the dN/dη value showed a stronger dependence on the working point. As one can see in the table, the working point defined this way reproduces the true value for the average transverse momentum in each case within a few percent as well. From model to model, different cuts are needed to reproduce the true values of the measured quantities in charge exchange events.

## 4. Conclusions

Our goal was to propose a method to efficiently select charge exchange events at the LHC using simple measurable quantities, and to motivate the LHC experiments to measure collision events that contain a highly energetic forward neutron. Those events can be selected using the zero degree calorimeters. With the help of some additional selections based on other detectors proposed here, those neutron events can be further cleaned from the inclusive background to approximate charge exchange events more accurately. By making comparisons between selected Monte Carlo events and experimental data to be measured in the future, one can differentiate between the relevant interaction models and determine which one provides the best predictions. By improving the precision of the predictions of these models at LHC energies, their predictions at cosmic ray energies might also improve, which was the major motivation of this work. For examining charge exchange processes and using the most commonly employed event generators, we suggest a specific event selection; we used the acceptances of some detectors in the CMS experiment as an example. When applying this method, namely, requiring a minimum of 2140 GeV energy deposited in the ZDC+, a minimal energy in any of the HFs and also a more than 102 GeV energy difference between HFs on different sides, we got the closest values to the true charge exchange events for pseudorapidity density and average transverse momentum of charged pions in the tracker, according to the EPOS 1.99 model. Other models gave somewhat different predictions for the optimal selection. We have also shown that taking into account the energy resolution of the ZDC deteriorates the purity of the event selection. The presented event selection, applied to Monte Carlo event generators as well as collision data to be collected by the LHC in the future, causes a significant improvement in the precision of the modeling of charge exchange events at the LHC, and therefore also in the modeling of cosmic rays’ physics. Improving the description of the pion production in pion–proton and pion–nucleus collisions can contribute to a better understanding of the muon puzzle in extended air showers of high energy cosmic rays.

## Figures and Tables

**Table 1:** Predictions for optimal working point cuts using the four models that can handle charge exchange. The event selection efficiency and purity values and the required minimal energy difference in the HFs are listed. The differences in percentage from the true value of the two measurable quantities after the cuts are also shown.

Quantity	EPOS 1.99	EPOS-LHC	PYTHIA	SIBYLL 2.3c	Average
**Efficiency [%]**	48.6	49.1	57.1	50.7	51.4 ± 5.7
**Purity [%]**	63.1	55.5	34.2	25.4	44.6 ± 18.6
**ΔEnergy [GeV]**	102.4	95.0	75.7	111.0	96.0 ± 14.9
**dN/d*η* vs. true [%]**	0.0	0.0	0.0	0.0	0.0 ± 0.0
**$\langle p_{T}\rangle$ vs. true [%]**	0.7	1.9	2.0	3.8	2.1 ± 1.7

## Data Availability

Not applicable.
